# Natural history of Type 1 spinal muscular atrophy: a retrospective, global, multicenter study

**DOI:** 10.1186/s13023-022-02455-x

**Published:** 2022-07-29

**Authors:** Claude Cances, Dmitry Vlodavets, Giacomo Pietro Comi, Riccardo Masson, Maria Mazurkiewicz-Bełdzińska, Kayoko Saito, Edmar Zanoteli, Angela Dodman, Muna El-Khairi, Ksenija Gorni, Isaac Gravestock, Janine Hoffart, Renata S. Scalco, Basil T. Darras, the ANCHOVY Working Group, Katia Alberti, Giovanni Baranello, Nina Barisic, Noemi Brolatti, Claudio Bruno, Claude Cances, Giacomo Pietro Comi, Basil T. Darras, Nicolas Deconinck, Elke Vos, Liesbeth De Waele, Angela Dodman, Claudia Dosi, Muna El-Khairi, Amanda Engelbrekt, Nathalie Goemans, Ksenija Gorni, Alessandra Govoni, Isaac Gravestock, Kazuhiro Haginoya, Janine Hoffart, Katarzyna Kotulska-Jozwiak, Laure Le Goff, Alexis Levine, Saidi Manel, Riccardo Masson, Chiara Mastella, Eleonora Mauri, Maria Mazurkiewicz-Bełdzińska, Megi Meneri, Isabella Moroni, Katarzyna Pierzchlewicz, Aurelie Portefaix, Alexandra Prufer, Myriam Rauso, Kayoko Saito, Renata S. Scalco, Veronica Schembri, Mariangela Sicolo, Valentine Tahon, Josipa Tomas, Dominique Vincent-Genod, Dmitry Vlodavets, Carole Vuillerot, Kazuyuki Yotsumata, Edmar Zanoteli

**Affiliations:** 1 AOC (Atlantic-Oceania-Caribbean) Reference Centre for Neuromuscular Disorders, Paediatric Clinical Research Unit/Paediatric Multi-Thematic Module CIC 1436, Neuropaediatric Department, Toulouse University Hospital, Toulouse, France; 2 Pediatric Clinical Research Unit, Pediatric Plurithematic Module, CIC 1436, Toulouse, France; 3 Russian Children Neuromuscular Center, Veltischev Clinical Pediatric Research Institute of Pirogov Russian National Research Medical University, Moscow, Russia; 4 Dino Ferrari Center, Department of Pathophysiology and Transplantation, University of Milan, Milan, Italy; 5 IRCCS Foundation Ca’ Granda Ospedale Maggiore Policlinico, Neuromuscular and Rare Diseases Unit, Milan, Italy; 6 Developmental Neurology Unit, Fondazione IRCCS Istituto Neurologico Carlo Besta, Milan, Italy; 7 Department of Developmental Neurology, Medical University of Gdańsk, Gdańsk, Poland; 8 Institute of Medical Genetics, Tokyo Women’s Medical University, Tokyo, Japan; 9 Department of Neurology, Faculdade de Medicina, Universidade de São Paulo (FMUSP), São Paulo, Brazil; 10 Pharma Development Neurology, F. Hoffmann-La Roche Ltd, Basel, Switzerland; 11 Roche Products Ltd, Welwyn Garden City, UK; 12 PDMA Neuroscience and Rare Disease, F. Hoffmann-La Roche Ltd, Basel, Switzerland; 13 Personalized Healthcare Analytics, F. Hoffmann-La Roche Ltd, Basel, Switzerland; 14 Department of Neurology, Boston Children’s Hospital, Harvard Medical School, Boston, MA USA

**Keywords:** ANCHOVY, FIREFISH, SMA natural history, Type 1 SMA, Spinal muscular atrophy

## Abstract

**Background:**

ANCHOVY was a global, multicenter, chart-review study that aimed to describe the natural history of Type 1 spinal muscular atrophy (SMA) from a broad geographical area and provide further contextualization of results from the FIREFISH (NCT02913482) interventional study of risdiplam treatment in Type 1 SMA.

**Methods:**

Data were extracted from medical records of patients with first symptoms attributable to Type 1 SMA between 28 days and 3 months of age, genetic confirmation of SMA, and confirmed survival of motor neuron 2 copy number of two or unknown. The study period started on 1 January 2008 for all sites; study end dates were site-specific due to local treatment availabilities. Primary endpoints were time to death and/or permanent ventilation and proportion of patients achieving motor milestones. Secondary endpoints included time to initiation of respiratory and feeding support.

**Results:**

Data for 60 patients from nine countries across Asia, Europe and North and South America were analyzed. The median age (interquartile range [IQR]) for reaching death or permanent ventilation was ~ 7.3 (5.9–10.5) months. The median age (IQR) at permanent ventilation was ~ 12.7 (6.9–16.4) months and at death was ~ 41.2 (7.3–not applicable) months. No patients were able to sit without support or achieved any level of crawling, standing or walking.

**Interpretation:**

Findings from ANCHOVY were consistent with published natural history data on Type 1 SMA demonstrating the disease’s devastating course, which markedly differed from risdiplam-treated infants (FIREFISH Part 2). The results provide meaningful additions to the literature, including a broader geographical representation.

**Supplementary Information:**

The online version contains supplementary material available at 10.1186/s13023-022-02455-x.

## Background

Spinal muscular atrophy (SMA) is a severe, progressive, neuromuscular disease, and was the leading genetic cause of infant mortality prior to the availability of current disease-modifying treatments [1, 2]. It is caused by loss of functional survival of motor neuron (SMN) protein due to genetic mutations or deletions of the *SMN1* gene [1, 3–5]. *SMN2* is a paralogous SMN gene that also encodes SMN protein; however, during splicing, exon 7 is excluded from the transcript, resulting in low levels of functional SMN protein [4, 5]. Prior to the availability of disease-modifying treatments, SMA subtypes were classified as Type 0 through 4 (most to least severe), based on age at onset and the most advanced motor milestone achieved [6, 7]. Recently, there has been movement to categorize patients with SMA by their functional ability rather than type [8]. *SMN2* copy number inversely correlates with SMA disease severity (i.e. patients with milder SMA phenotypes typically have higher *SMN2* copy numbers than patients with more severe SMA types) [7].

Type 1 SMA is characterized by symptom onset before 6 months of age, the inability to sit without support (i.e. non-sitters), and is usually associated with two copies of *SMN2* [1, 9–12]. These infants, if untreated, rarely achieve any developmental motor milestones and typically die before 2 years of age [1]. Clinical features of Type 1 SMA predominantly arise from neuromuscular weakness. Infants with Type 1 SMA exhibit weakened limbs; weakness of intercostal muscles that leads to a paradoxical breathing pattern, weak cough and respiratory insufficiency; and bulbar motor neuron involvement that leads to swallowing and feeding difficulties [13, 14]. These infants require feeding support or combined feeding and ventilatory support by 12 months of age [10].

Patients with Type 1 SMA have complex needs and require a multidisciplinary approach to care, which includes neuromuscular and musculoskeletal, rehabilitation, orthopedic, nutritional, swallowing, gastrointestinal and pulmonary management [15, 16]. To date, three disease-modifying therapies have been approved for the treatment of SMA, including Type 1 SMA (therapeutic indications are specified in the respective drug labels): the orally administered *SMN2* pre-mRNA splicing modifier risdiplam (EVRYSDI®) [17–20]; the single-dose, intravenously administered adeno-associated virus 9 *SMN1* gene replacement therapy onasemnogene abeparvovec (ZOLGENSMA®) [21, 22]; and the intrathecally administered *SMN2*-directed antisense oligonucleotide nusinersen (SPINRAZA®) [23, 24]. In clinical studies, these three therapies have demonstrated increased survival and improvements in motor function in infants with Type 1 SMA [25–28].

Advancements in standard of care (SOC) over the past decade have led to an improvement in the natural history of Type 1 SMA, such as increased survival rates and better quality of life [10, 15, 16, 29, 30]. Disease progression of Type 1 SMA has been well described in natural history studies; however, these studies report limited data on some aspects of disease progression (such as abnormal swallowing), some were conducted prior to advances in SOC, and most present data from a single country [10–12, 30–32].

The ANCHOVY study was a global, multicenter, chart-review study that provided an update on natural history data in patients with Type 1 SMA from a broad geographical area. The study aimed to describe the natural history of a patient population that was similar to the population in the FIREFISH study (NCT02913482), an open-label, two-part study of risdiplam in symptomatic infants with Type 1 SMA and two *SMN2* gene copies [27, 28], and who had received similar SOC. Here, we first present the findings of the ANCHOVY study and then compare these natural history results with results from Part 2 of the FIREFISH study.

## Results

### ANCHOVY

#### Patients

A total of 60 patients with Type 1 SMA were analyzed in the ANCHOVY study (Additional file 1: Fig. S1). Key patient demographics and characteristics are summarized in Table 1. The median age at onset of SMA symptoms was 1.6 (range 1.0–3.0) months. Half of the patients were reported to have two *SMN2* copies, while *SMN2* copy number for the remaining half was unknown. Hypotonia was the most frequently reported initial symptom (97%) followed by absent deep tendon reflexes (77%) and limb weakness (65%) (Table 2).

**Table 1:** Key patient demographics and characteristics: ANCHOVY and FIREFISH Part 2

	ANCHOVY patients	FIREFISH Part 2 patients
	(N = 60)	(N = 41)
Age at SMA symptom onset, months, median (range)	1.6 (1.0–3.0)	1.5 (1.0–3.0)
*Sex, n (%)*		
Male	39 (65)	19 (46)
Female	21 (35)	22 (54)
*Country, n (%)*		
Belgium	5 (8)	0
Brazil	6 (10)	3 (7)
China	0	11 (27)
Croatia	3 (5)	1 (2)
France	10 (17)	4 (10)
Italy	10 (17)	10 (24)
Japan	7 (12)	1 (2)
Poland	4 (7)	4 (10)
Russia	8 (13)	5 (12)
Turkey	0	1 (2)
USA	7 (12)	1 (2)
*Confirmed SMN2 copy number, n (%)*		
Two	30 (50)	41 (100)*
Unknown	30 (50)	0

*Patients in FIREFISH were required to have two copies of *SMN2* per the inclusion criteria

SMA, spinal muscular atrophy; *SMN2*, survival of motor neuron 2

**Table 2:** Initial SMA symptoms reported: ANCHOVY

Initial symptoms, n (%)	ANCHOVY patients (N = 60)
Hypotonia	58 (97)
Absent deep tendon reflexes	46 (77)
Limb weakness	39 (65)
Developmental motor delay	38 (63)
Inability to sit independently	38 (63)
Tongue fasciculations	31 (52)
Swallowing/feeding difficulties	22 (37)
Pneumonia/respiratory symptoms	20 (33)

SMA, spinal muscular atrophy

#### Primary endpoints

##### Event-free survival

**Fig. 1:**
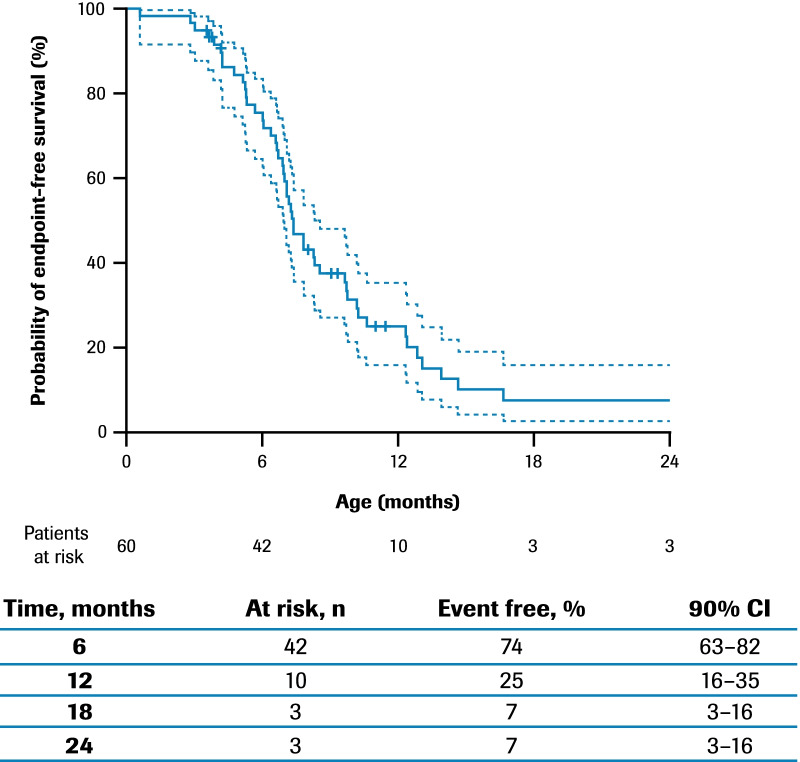
Time to death or permanent ventilation*: ANCHOVY. *Permanent ventilation was defined as ≥ 16 h of non-invasive ventilation per day for > 21 consecutive days, intubation for > 21 consecutive days, or tracheostomy. 90% CIs are calculated with a complementary log–log transformation for the estimated survival function, with standard errors computed via Greenwood’s formula. Patients with no recorded event are censored at the last age they were known to be event free. Two additional patients died after 24 months of age. CI, confidence interval

The median age (interquartile range [IQR]) for reaching death or permanent ventilation was ~ 7.3 (5.9–10.5) months (Fig. 1). The median age (IQR) for reaching permanent ventilation was ~ 12.7 (6.9–16.4) months (Additional file 1: Fig. S2) and for death was ~ 41.2 (7.3–not applicable) months (Additional file 1: Fig. S3).

##### Motor milestones (Hammersmith Infant Neurological Examination, Section 2)

At 12 months of age, no patients with data available (n = 9) achieved any level of sitting or head control (Table 3). Some head control was achieved up to 9 months of age: 11 observations of partial head control “wobbles” were achieved in eight patients (four of these patients had two *SMN2* copies and four patients had unknown *SMN2* copy number), with some patients achieving the milestone at multiple time points, and one patient (unknown *SMN2* copy number) was able to maintain upright head control at 6 months of age. At 9 months of age, one patient (with two *SMN2* copies) was able to sit with support. No patients achieved sitting without support at any time point. Rolling (to the side) was achieved in two patients up to 6 months of age, with one patient achieving the milestone at two time points. At 12 months of age, no Hammersmith Infant Neurological Examination, Section 2 (HINE-2) motor milestones were achieved in voluntary grasp, kicking, or rolling among the patients who had milestone data (n = 8; n = 9; n = 9, respectively) (Additional file 1: Table S1). Patients did not achieve any level of crawling, standing, or walking at any time point.

**Table 3:** Sitting ability and head control assessed by HINE-2: ANCHOVY

	M3 (N = 60)	M6 (N = 60)	M9 (N = 60)	M12 (N = 60)	M15 (N = 60)	M18 (N = 60)	M21 (N = 60)	M24 (N = 60)
*Sitting, n (%)*								
0: Cannot sit	25 (42)	19 (32)	8 (13)	9 (15)	5 (8)	5 (8)	4 (7)	5 (8)
1: Sits with support at hips	0	0	1 (2)	0	0	0	0	0
2: Props	0	0	0	0	0	0	0	0
3: Stable sit	0	0	0	0	0	0	0	0
4: Pivots (rotates)	0	0	0	0	0	0	0	0
Not recorded in chart*	5 (8)	3 (5)	0	0	0	0	0	0
*Head control, n (%)*								
0: Unable to maintain upright	26 (43)	14 (23)	8 (13)	9 (15)	5 (8)	5 (8)	4 (7)	5 (8)
1: Wobbles^†^	4 (7)	6 (10)	1 (2)	0	0	0	0	0
2: All the time upright	0	1 (2)	0	0	0	0	0	0
Not recorded in chart*	0	1 (2)	0	0	0	0	0	0
Missing^‡,§^	30 (50)	34 (57)	35 (58)	26 (43)	27 (45)	27 (45)	28 (47)	27 (45)
Censored^‡,¶^	0	1 (2)	1 (2)	2 (3)	2 (3)	2 (3)	2 (3)	2 (3)
Death^‡,^**	0	3 (5)	15 (25)	23 (38)	26 (43)	26 (43)	26 (43)	26 (43)

There was no consistent assessment schedule that patients followed during the course of medical care and as such, most time points have sparse data for HINE-2 assessments. No patients achieved any level of sitting or head control after 24 months of age

*Hospital record exists at this time, but no mention of this motor milestone

^†^The 11 observations of wobbles were reported for eight patients overall, with some patients achieving this milestone at multiple time points

^‡^Applies to both sitting and head control data

^§^No records for this time period

^¶^Patient was excluded from study at time points after starting treatment (nusinersen) or after enrollment in a clinical trial

**Patient died before the beginning of this time period

HINE-2, Hammersmith Infant Neurological Examination, Section 2; M, month

Many patients had missing data for the HINE-2 assessments; 48% of patients (29/60) had only a single HINE-2 assessment. Notably, 22% of patients (13/60) had recorded sitting assessments at multiple time points; however, 43% (26/60) had only one recorded assessment of sitting and 35% (21/60) had no recorded assessment of sitting.

#### Secondary endpoints

##### Initiation of respiratory support

**Fig. 2:**
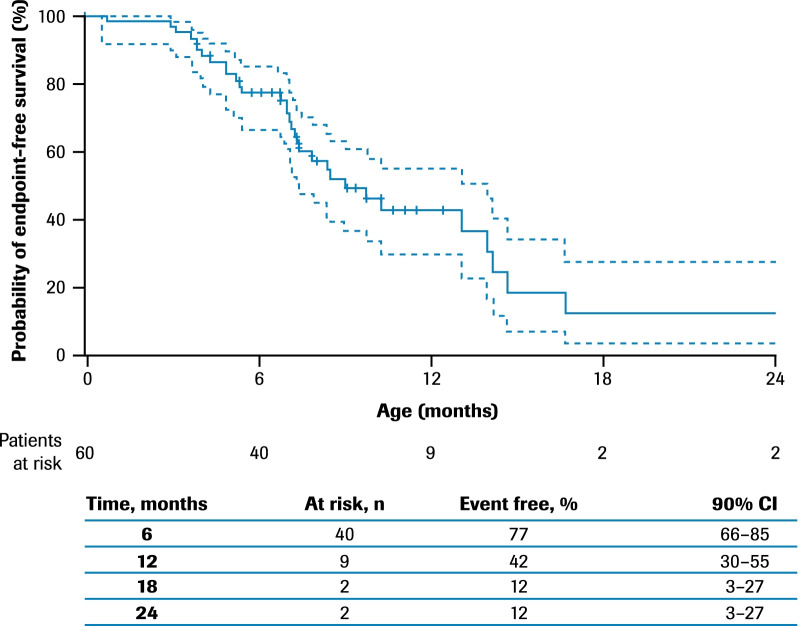
Time to respiratory support including permanent ventilation*: ANCHOVY. *Time from birth to first occurrence of awake-assisted, night-time-assisted, or nap-time-assisted ventilation, airway clearance through cough assistance or permanent ventilation (defined as ≥ 16 h of non-invasive ventilation per day for > 21 consecutive days, intubation for > 21 consecutive days, or tracheostomy). 90% CIs are calculated with a complementary log–log transformation for the estimated survival function, with standard errors computed via Greenwood’s formula. Patients with no recorded events are censored at the last age they were known to be event free. One additional patient required respiratory support after 24 months of age. CI, confidence interval

The median age (IQR) at initiation of respiratory support was ~ 8.8 (6.8–13.9) months (Fig. 2).

##### Swallowing and feeding support

**Fig. 3:**
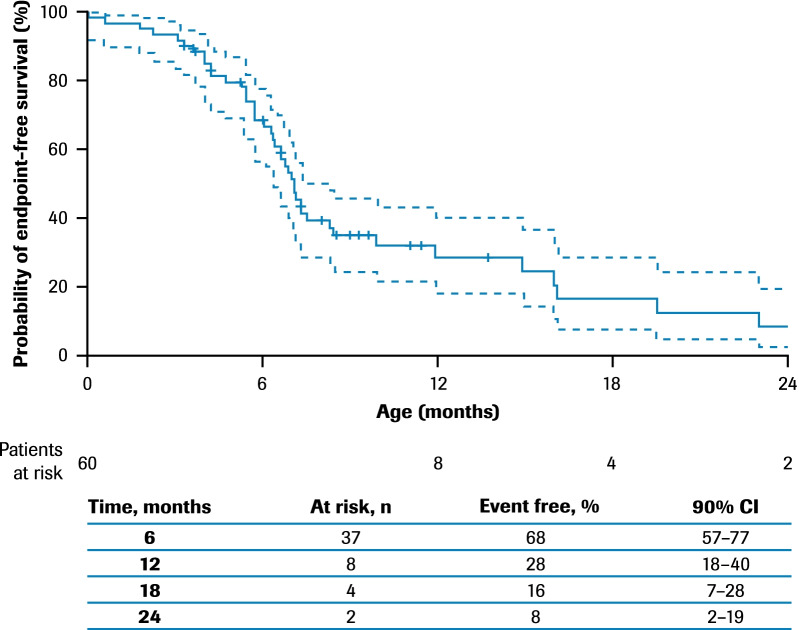
Time to feeding support*: ANCHOVY. *Feeding support included placement of nasogastric or nasojejunal tube or gastrostomy. 90% CIs are calculated with a complementary log–log transformation for the estimated survival function, with standard errors computed via Greenwood’s formula. Patients with no recorded events are censored at the last age they were known to be event free. There were no additional events recorded after 24 months of age. CI, confidence interval

Abnormal swallowing was observed: the median age (IQR) at onset was ~ 6.6 (5.3–12.1) months (Additional file 1: Fig. S4) and at initiation of feeding support was ~ 6.9 (5.3–14.7) months (Fig. 3). At 12 months of age, there were 25 patients who were alive and had nutritional support data available; of these, 76% (19/25) required feeding support via a feeding tube.

##### Anthropometric data

Among all patients who were alive and had growth measurements recorded in the 3 months of age window (n = 22 for length/height; n = 23 for weight), the median length/height-for-age value (range) was the 26th (0–100th) percentile and the median weight-for-age value (range) was the 21st (0–83rd) percentile, according to World Health Organization growth charts [33, 34]. Growth measurement data are presented for the 3-month age window as beyond this window most data were missing (see Additional file 1: Fig. S5A and S5B for all available growth measurement data).

### Comparisons of ANCHOVY and FIREFISH Part 2

Baseline demographics and disease characteristics were comparable between the ANCHOVY and FIREFISH Part 2 (N = 41) populations (Table 1). The median age at onset of SMA symptoms in the FIREFISH Part 2 population was 1.5 (range 1.0–3.0) months [28]. In FIREFISH Part 2, all patients had two copies of *SMN2*, as specified in the inclusion criteria. In both studies, the majority of patients were enrolled in Europe or the USA; however, one difference between the two studies was that no patients from China were included in the ANCHOVY study, as approval timelines were not aligned with the global study timelines.

#### Survival and event-free survival

In FIREFISH Part 2, 93% (90% confidence interval [CI] 82–97%) of infants were alive and 85% (90% CI 73–92%) of infants were alive without the need for permanent ventilation following treatment with risdiplam for 12 months (age range 14.5–18.9 months) [28]. In ANCHOVY, 51% (90% CI 32–62%) of patients were alive and 7% (90% CI 3–16%) of patients were alive without the need for permanent ventilation at 18 months of age (Fig. 1).

**Fig. 4:**
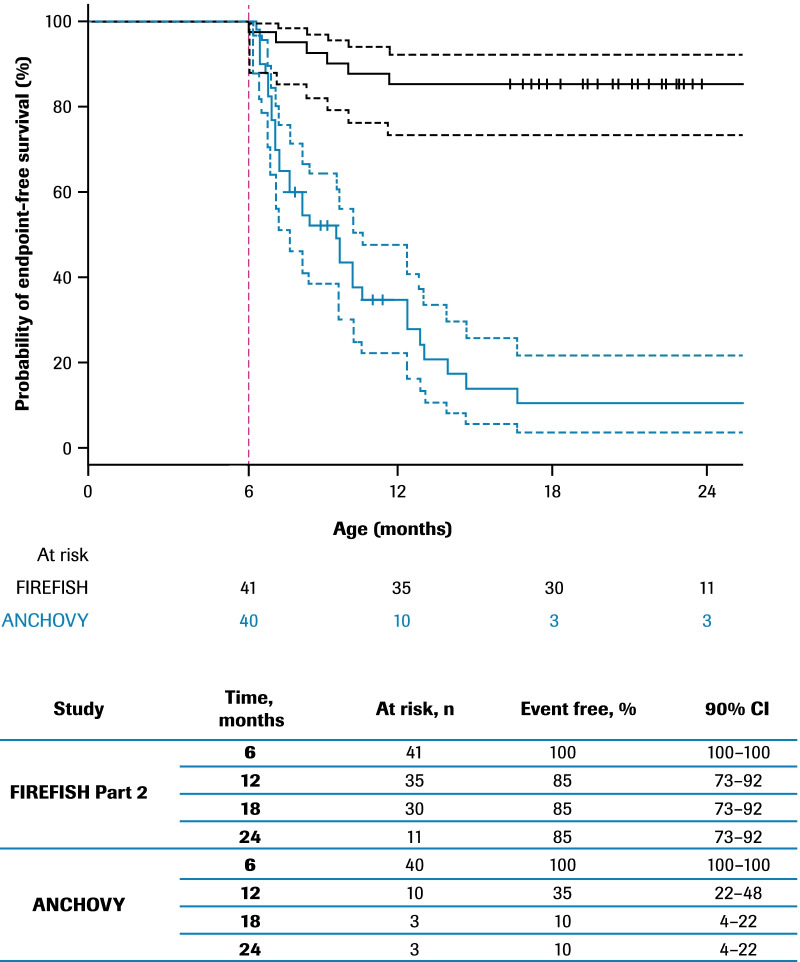
Landmark comparison of time to death or permanent ventilation: ANCHOVY and FIREFISH Part 2. Kaplan–Meier curves are shown for FIREFISH (n = 41; solid black) and ANCHOVY (n = 40; solid blue). To compensate for the differences in age at the start of the risk period in the FIREFISH Part 2 and ANCHOVY studies, the landmark (dotted red vertical line) was set at the youngest age that a patient had an event in the FIREFISH Part 2 study, which was at an age of 6.1 months (186 days). ANCHOVY patients who had events before this time point are excluded. CI, confidence interval

A landmark analysis of event-free survival (as described in the Statistical Methods) was performed to compensate for the immortal time bias between ANCHOVY and FIREFISH Part 2, which was due to the fact that some patients in the ANCHOVY study had events before patients were at risk for an event in the FIREFISH study. Starting the analysis at a landmark age allowed for a comparison of event-free survival between patients in FIREFISH Part 2 and a subset of ANCHOVY patients who were event free up until the age of the youngest patient with an event in FIREFISH Part 2. The first event of death or permanent ventilation in FIREFISH Part 2, defining the landmark age, occurred at an age of 6.1 months (186 days). At the landmark, 16 patients from the ANCHOVY study already had events of death or permanent ventilation and four were censored; hence, 20 patients from ANCHOVY were excluded from the landmark analysis and 40 were included. No FIREFISH patients were excluded from the analysis. In the landmark analysis, 10% (90% CI 4–22%) of infants in ANCHOVY were alive without the need for permanent ventilation at 18 months of age compared with 85% (90% CI 73–92%) in FIREFISH Part 2 (Fig. 4).

#### Motor milestones (Hammersmith Infant Neurological Examination, Section 2)

No patients in the ANCHOVY study with an assessment for the sitting and head control milestones (n = 5) were reported to sit without support or were able to maintain upright head control at 18 months of age, compared with 24% (10/41) of infants achieving sitting without support (stable sits or pivots [rotates]) and 44% (18/41) of infants able to maintain upright head control in the FIREFISH Part 2 study after 12 months of risdiplam treatment (Table 4) [28].

**Table 4:** Sitting ability and head control assessed by HINE-2: ANCHOVY and FIREFISH Part 2

HINE-2 motor milestone	All ANCHOVY patients 18 months of age (N = 60)	All FIREFISH Part 2 patients 12 months of risdiplam treatment (N = 41)
*Sitting, n (%)*		
Stable sit or pivots (rotates)	0	10 (24)
Sits with support at hips or props	0	15 (37)
Cannot sit	5 (8)	13 (32)
Not recorded in chart*	0	0
*Head control, n (%)*		
All the time upright	0	18 (44)
Wobbles	0	13 (32)
Unable to maintain upright	5 (8)	7 (17)
Not recorded in chart*	0	0
Missing^†,‡^	27 (45)	0
Censored^†,§^	2 (3)	0
Death^†,¶^	26 (43)	3 (7)

Able to sit is defined as achieving level 3 or 4 of HINE-2

*Hospital record exists at this time, but no mention of this motor milestone

^†^Applies to both sitting and head control data

^‡^No records for this time period

^§^Patient was excluded from study at time points after starting treatment (nusinersen) or after enrollment in a clinical trial

^¶^Patient died before the beginning of this time period

HINE-2, Hammersmith Infant Neurological Examination, Section 2

#### Feeding support

Of the 23 patients who were alive and had nutritional support data available in ANCHOVY at 18 months of age, 87% (20/23) required feeding support via a feeding tube. In comparison, among the 38 patients in FIREFISH Part 2 who were alive following 12 months of treatment, 26% (10/38) received feeding support and 74% (28/38) were fed exclusively orally.

## Discussion

Our findings demonstrate that data from the ANCHOVY study are consistent with data reported in previous natural history studies of Type 1 SMA [10, 11, 31]. Although there are some differences in the definitions of feeding/nutritional support and respiratory/ventilation support between the ANCHOVY study and previously published natural history studies, the times to reaching these events were similar.

The median age for reaching death or permanent ventilation of ~ 7.3 months in the ANCHOVY study was similar to survival rates reported in the NeuroNEXT and Pediatric Neuromuscular Clinical Research (PNCR) Network natural history studies [10, 11]. In the NeuroNEXT study, conducted at 14 sites in the USA, the median age for reaching death or endotracheal intubation for infants with Type 1 SMA with two or unknown number of *SMN2* copies (n = 20) was 8 months [11]. In the PNCR study, conducted at three centers in the USA, the median age for reaching death or permanent ventilation (defined as requiring at least 16 h/day of non-invasive ventilation support for at least 14 days) in infants with two *SMN2* copies only (n = 23) was 10.5 months [10]. Furthermore, data on motor milestone achievement from the ANCHOVY study were consistent with data from a natural history study; no patients with Type 1 SMA were able to sit without support or achieved any level of crawling, standing, or walking as assessed by the HINE-2, while some patients showed some level of head control, kicking, and hand grasp [31].

In the PNCR study, the median ages (IQR) at initiation of nutritional support (placement of nasogastric or gastrostomy tube) and ventilatory support (non-invasive ventilation or intubation leading to tracheostomy) for infants with Type 1 SMA were 8 (6–13) months and 11 (5–19) months, respectively; all patients aged greater than 12 months at baseline required feeding support or combined feeding and ventilatory support [10]. Similarly, in the ANCHOVY study, the median ages (IQR) at initiation of feeding support and respiratory support were ~ 6.9 (5.3–14.7) months and ~ 8.8 (6.8–13.9) months, respectively; of the 25 patients alive and with nutritional support data at 12 months of age, 76% (19/25) required feeding support.

Comparing the key baseline and SMA disease characteristics for ANCHOVY and FIREFISH Part 2 [28] demonstrated that the populations are similar. Although the *SMN2* copy number for half of the patients in ANCHOVY was unknown, based on age of onset of symptoms and disease progression, these patients were classified as having Type 1 SMA. In the landmark analysis, at 18 months of age, the proportion of patients alive without permanent ventilation was 10% for ANCHOVY compared with 85% for FIREFISH Part 2. Even after conservatively accounting for the immortal time bias with the landmark analysis, this great disparity demonstrates the robust differentiation of event-free survival between the two studies. Notably, 51% (90% CI 39–62%) of infants in the ANCHOVY study were alive at 18 months of age, compared with 93% (90% CI 82–97%) in FIREFISH Part 2 after 12 months of risdiplam treatment [28].

In the ANCHOVY study, no patient with an assessment could achieve any level of sitting at 18 months of age, as assessed by the HINE-2. In comparison, 61% (25/41) of infants achieved some level of sitting in FIREFISH Part 2, with 24% (10/41) achieving sitting without support (six patients [15%] achieved a stable sit and four patients [10%] were able to pivot [rotate] while sitting) after 12 months of treatment. While demographic and baseline disease characteristics were comparable between the ANCHOVY and FIREFISH Part 2 studies, the marked difference in event-free survival, achievement of motor milestones and initiation of feeding support for FIREFISH Part 2 participants compared with ANCHOVY patients further supports the benefit of risdiplam in patients with Type 1 SMA.

This study was limited due to its retrospective design, resulting in missing information (as the original data were not collected according to a research protocol) and potential bias due to factors such as differences in baseline characteristics, subject selection and unknown loss of follow-up. Furthermore, motor milestone assessments may not have been conducted as the disease progressed and patients’ overall health declined. For example, 78% of patients had only one or no recorded sitting assessment; therefore, it is difficult to interpret the longitudinal data. Additionally, with no age limit for first consultation and patients being followed until death or last medical visit recorded in the chart, the length of patient follow-up varied widely.

## Conclusions

Findings from the ANCHOVY study were consistent with the published natural history data on Type 1 SMA, demonstrating the devastating course of this disease. Untreated infants with Type 1 SMA in ANCHOVY reached death or permanent ventilation, required feeding/nutritional support and respiratory/ventilatory support at time points comparable to those reported in other natural history studies. Similarly, they did not achieve or retain most motor milestones, which is consistent with historical cohorts. These outcomes markedly contrasted with those achieved by risdiplam-treated infants in FIREFISH Part 2. Overall, ANCHOVY provides meaningful additions to previously published natural history studies by including data from countries outside Europe and the USA, such as Brazil and Russia, that had not previously been represented.

## Methods

### Study design and analysis population

ANCHOVY was a retrospective cohort study of patients with Type 1 SMA treated in the same centers as patients in the FIREFISH study or in a center in the same country that provided similar SOC, as confirmed by FIREFISH Principal Investigators. Data on SOC approaches in each country were not collected. Participants were enrolled if the first signs or symptoms attributable to Type 1 SMA (including hypotonia, absent deep tendon reflexes and/or tongue fasciculations) occurred between 28 days and 3 months of age, and there was a genetic confirmation of homozygous deletion or compound heterozygosity predictive of loss of function of the *SMN1* gene, and two confirmed *SMN2* copies or unknown copy number.

The study was carried out in 18 sites across nine countries (Belgium, Brazil, Croatia, France, Italy, Japan, Poland, Russia and the USA). For all sites, the start of the study period was 1 January 2008. The inclusion period end date was specific to each study site to ensure that included patients were representative of the source population and did not over-represent patients who were ineligible or whose parents/caregivers were unwilling to have their child enter a clinical trial, or who had received an approved SMA therapy. For sites that participated in any investigational drug study in Type 1 SMA, including FIREFISH, the study period ended 7 months prior to the first patient enrolled in an investigational drug study at the site. For sites that did not participate in any investigational drug study in Type 1 SMA, the study period ended 12 months prior to the first availability (either through an early-access program or commercially) of nusinersen at the site. Only patients whose first visit at the study site occurred during the site-specific study period and who met the above-mentioned patient population criteria were included in the analysis. The study population and inclusion period for ANCHOVY were defined to include patients who had comparable demographics, disease characteristics and SOC to patients included in the FIREFISH study.

### Study outcomes

The primary endpoints for this study were time to death, time to permanent ventilation (defined as requiring at least 16 h of non-invasive ventilation per day for more than 21 consecutive days, intubation for more than 21 consecutive days, or tracheostomy), the composite endpoint of time to death or permanent ventilation (the date of the event that occurred first was used), and the proportion of patients who achieved motor milestones (head control, voluntary grasp, kicking, rolling, crawling, sitting, standing, and walking) assessed by the HINE-2, a standardized tool for specifically measuring infant developmental motor milestones [31, 35, 36].

Secondary endpoints included time to use of respiratory support (defined as the first occurrence of awake-assisted, night-time-assisted or nap-time-assisted ventilation, airway clearance through cough assistance or permanent ventilation), time to onset of abnormal swallowing (defined as the first event of abnormal swallowing as determined by clinical measurement [if available], clinician assessment, or parent/caregiver report), time to initiation of feeding support (placement of nasogastric or nasojejunal tube or gastrostomy) and anthropometric measurements (length/height and weight). The full list of secondary endpoints is provided in Additional file 1. For all time-to-event analyses, patients with no event recorded in the charts were censored at the last age they were known to be event free.

Additional analyses that were not pre-specified in the ANCHOVY statistical analysis plan were carried out to compare data from the ANCHOVY and FIREFISH Part 2 [28] studies, including baseline characteristics, time to death or permanent ventilation, sitting and head control abilities assessed by the HINE-2, and initiation of feeding support.

Data are here presented with a focus on results at 18 months of age in ANCHOVY; this corresponds to the comparative age from the FIREFISH study by which patients had received 12 months of treatment with risdiplam (the median age at enrollment in FIREFISH Part 2 was 5.3 months) [28].

### Data collection and analysis

Patient data were extracted from medical records and recorded onto an electronic case report form by the physician or other qualified member of the clinical or research team. All medical charts available at site for the study period and meeting the study eligibility criteria were extracted. Data extraction variables included baseline demographics and disease characteristics, death, permanent ventilation, use of respiratory support, use of feeding support, anthropometric measurements, and motor function assessments.

### Statistical methods

Descriptive statistics were used to summarize the extracted data. All analyses were performed using the software R [37]. The primary analysis population included all patients who met the eligibility criteria of the study; this population was also used for the comparison with the FIREFISH Part 2 study data. Missing data were not imputed if not stated otherwise. The numbers of patients with missing data were reported for the HINE-2 assessments. Motor function and anthropometric data were summarized in 3-month age windows centered around the nominal age. For example, the Month 3 window was from 1.5 to 4.5 months of age.

For the comparison of time to death or permanent ventilation between ANCHOVY and FIREFISH, a sensitivity analysis was performed, herein referred to as the ‘landmark analysis’. This analysis compensates for the differences in age at the start of the risk periods in each study. A time point was designated as the ‘landmark age’ and only patients who survived until the landmark age were analyzed. The landmark age was set at the youngest age that an infant had an event in FIREFISH Part 2. ANCHOVY data used in the landmark analysis included only patients who were event free at the landmark age.

## Supplementary Information

**Additional file 1:** Secondary endpoints: Full list of ANCHOVY study secondary endpoints. **Fig. S1**. Patient flow diagram: ANCHOVY. **Fig. S2**. Time to permanent ventilation: ANCHOVY. Kaplan−Meier diagram illustrating probability of patients requiring permanent ventilation by up to 24 months of age. **Fig. S3**. Time to death: ANCHOVY. Kaplan−Meier diagram illustrating probability of patients dying by up to 24 months of age. **Fig. S4**. Time to abnormal swallowing: ANCHOVY. Kaplan−Meier diagram illustrating probability of onset of abnormal swallowing by up to 24 months of age. **Fig. S5**. Height and weight: ANCHOVY. Graphs illustrating height and weight measurements up to 48 months of age. **Table S1**. Other HINE-2 motor milestones: ANCHOVY. Table listing the numbers of patients achieving the following HINE-2 motor milestones at 3-monthly windows up to 24 months of age: voluntary grasp, kicking, rolling crawling, standing and walking.

## Data Availability

The datasets generated and analyzed during the current study are not publicly available due to their sensitive medical nature and the risk or reidentification. Reasonable requests for aggregate data may be made to global.scientific-communications@roche.com.

## Abbreviations

CI: Confidence interval
HINE-2: Hammersmith Infant Neurological Examination, Section 2
IQR: Interquartile range
PNCR: Pediatric Neuromuscular Clinical Research
SMA: Spinal muscular atrophy
SMN: Survival of motor neuron
SOC: Standard of care
