# Supplementary material for: Natural history of Type 1 spinal muscular atrophy: a retrospective, global, multicenter study
Source: Orphanet J Rare Dis. 2022 Jul 29;17:300. doi: 10.1186/s13023-022-02455-x (PMC9336055; doi:10.1186/s13023-022-02455-x)
Supplement: Supplementary file 1 — Additional file 1: Secondary endpoints: Full list of ANCHOVY study secondary endpoints. Fig. S1. Patient flow diagram: ANCHOVY. Fig. S2. Time to permanent ventilation: ANCHOVY. Kaplan−Meier diagram illustrating probability of patients requiring permanent ventilation by up to 24 months of age. Fig. S3. Time to death: ANCHOVY. Kaplan−Meier diagram illustrating probability of patients dying by up to 24 months of age. Fig. S4. Time to abnormal swallowing: ANCHOVY. Kaplan−Meier diagram illustrating probability of onset of abnormal swallowing by up to 24 months of age. Fig. S5. Height and weight: ANCHOVY. Graphs illustrating height and weight measurements up to 48 months of age. Table S1. Other HINE-2 motor milestones: ANCHOVY. Table listing the numbers of patients achieving the following HINE-2 motor milestones at 3-monthly windows up to 24 months of age: voluntary grasp, kicking, rolling crawling, standing and walking. [file 13023_2022_2455_MOESM1_ESM.pdf]

Natural History of Type 1 Spinal Muscular Atrophy: A Retrospective, Global, Multicenter Study

Additional file 1

Study outcomes....2
Secondary endpoints....2
Figures....3
Fig. S1. Patient flow diagram: ANCHOVY....3
Fig. S2. Time to permanent ventilation*: ANCHOVY....4
Fig. S3. Time to death: ANCHOVY....5
Fig. S4. Time to abnormal swallowing: ANCHOVY....6
Fig. S5. Height and weight: ANCHOVY....7
Table....8
Table S1. Other HINE-2 motor milestones: ANCHOVY....8

Study outcomes

Secondary endpoints

The secondary endpoints of this study were:

- Demographic characteristics including: age at time of Type 1 spinal muscular atrophy (SMA) diagnosis, sex, race/ethnicity (where permitted), initial SMA symptoms and family history of SMA
- Summary of total Hammersmith Infant Neurological Examination, Section 2 (HINE-2) score as derived from the following motor milestones: head control, voluntary grasp, kicking, rolling, crawling, sitting, standing and walking
- Proportion of patients with the ability to move their arms horizontally on a surface, thrust their arms and hold head in midline
- Growth measurements (head circumference, chest circumference, length/height and weight)
- Time to use of respiratory-support interventions (such as awake-assisted ventilation, night-time-assisted ventilation, nap-time-assisted ventilation, airway clearance through cough assistance and permanent ventilation)
- Time to first serious respiratory infection (i.e. requiring hospitalization or intravenous antibiotics)
- Rate of serious respiratory infections
- Time to onset of abnormal swallowing and to initiation of feeding support such as nasogastric tube or gastrostomy
- Overall rate of hospitalization

Figures

Fig. S1. Patient flow diagram: ANCHOVY

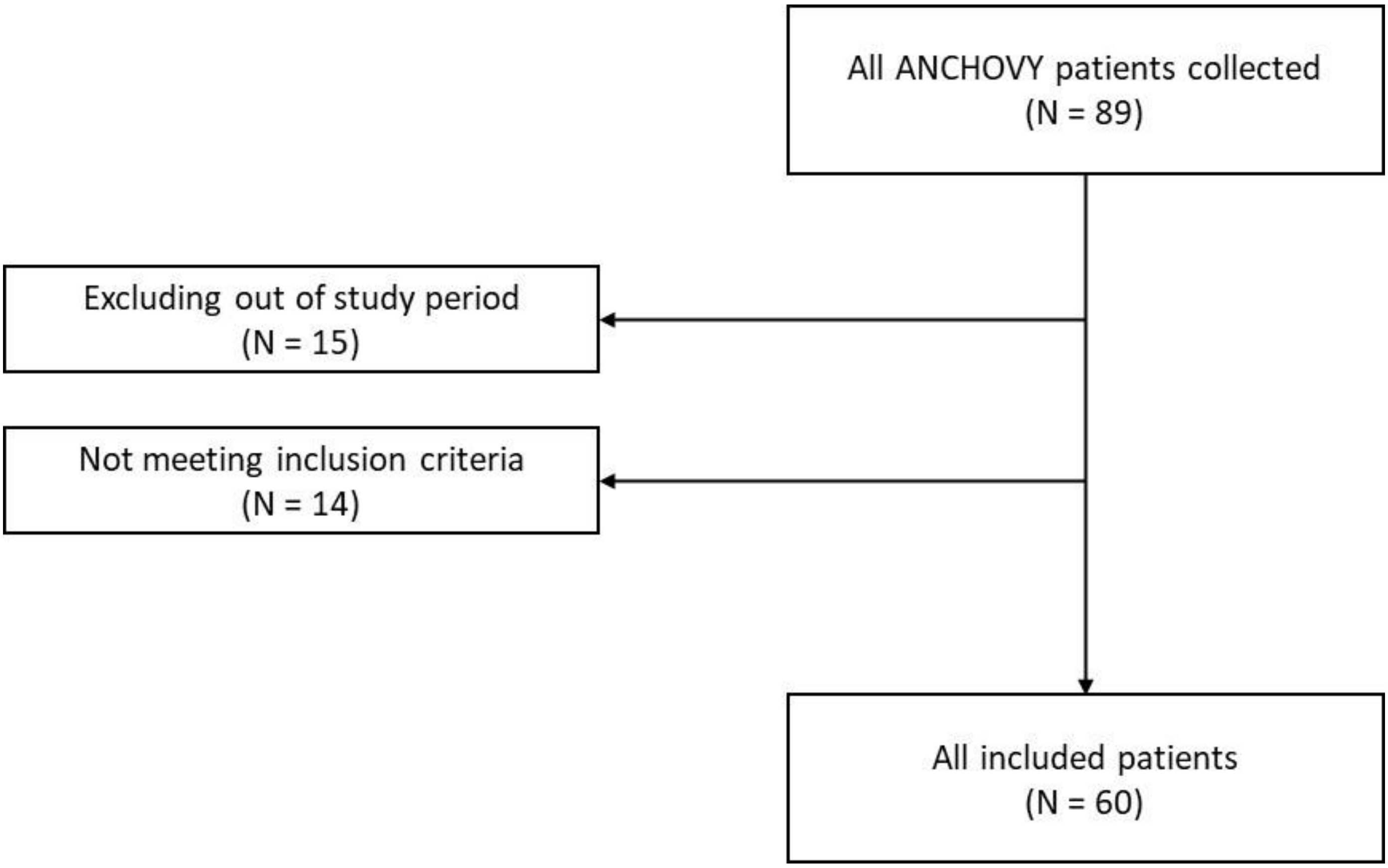

Fig. S2. Time to permanent ventilation*: ANCHOVY

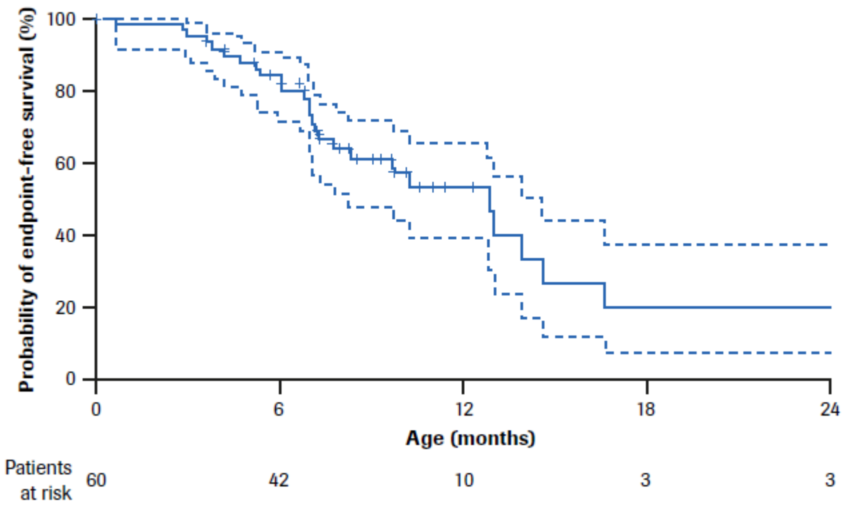

Time, months	At risk, n	Event free, %	90% CI
**6**	42	82	71–89
**12**	10	53	39–65
**18**	3	20	7–37
**24**	3	20	7–37

*Permanent ventilation was defined as ≥ 16 hours of non-invasive ventilation per day for > 21 consecutive days, intubation for > 21 consecutive days, or tracheostomy. 90% CIs are calculated with a complementary log–log transformation for the estimated survival function, with standard errors computed via Greenwood's formula. Patients with no recorded event are censored at the last age they were known to be event free. There were no additional events recorded after 24 months of age.

CI, confidence interval.

Fig. S3. Time to death: ANCHOVY

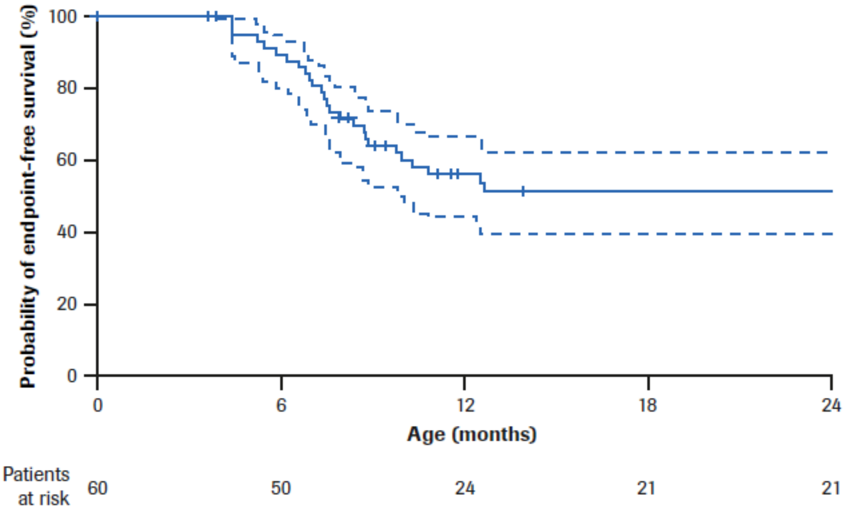

Time, months	At risk, n	Event free, %	90% CI
**6**	50	89	80–94
**12**	24	56	44–66
**18**	21	51	39–62
**24**	21	51	39–62

90% CIs are calculated with a complementary log−log transformation for the estimated survival function, with standard errors computed via Greenwood’s formula. Patients with no recorded events are censored at the last age they were known to be event free. Two additional patients died after 24 months of age.

CI, confidence interval.

Fig. S4. Time to abnormal swallowing: ANCHOVY

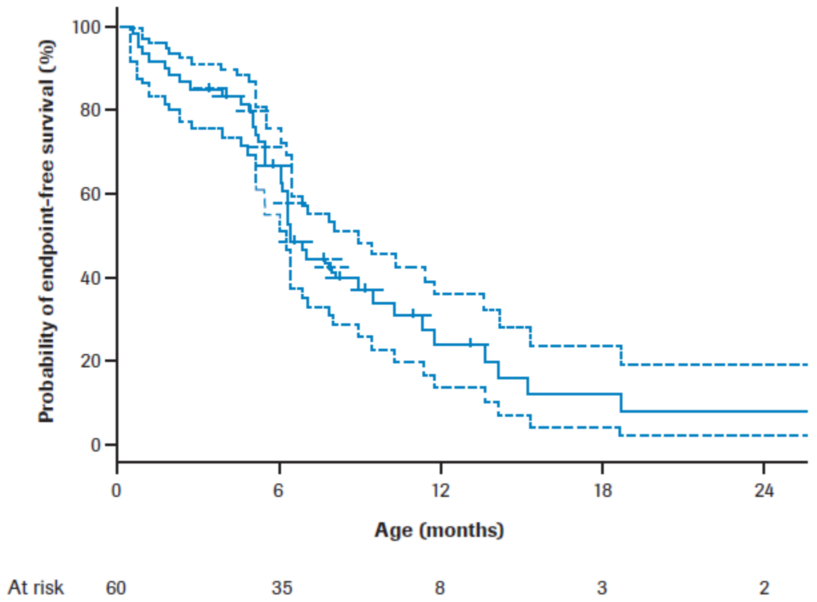

Time, months	At risk, n	Event free, %	90% CI
**6**	35	67	55–76
**12**	8	27	17–39
**18**	3	12	4–24
**24**	2	8	2–19

90% CIs are calculated with a complementary log−log transformation for the estimated survival function, with standard errors computed via Greenwood's formula. Patients with no recorded event are censored at the last age they were known to be event free. There were no additional events recorded after 24 months of age.

CI, confidence interval.

Fig. S5. Height and weight: ANCHOVY

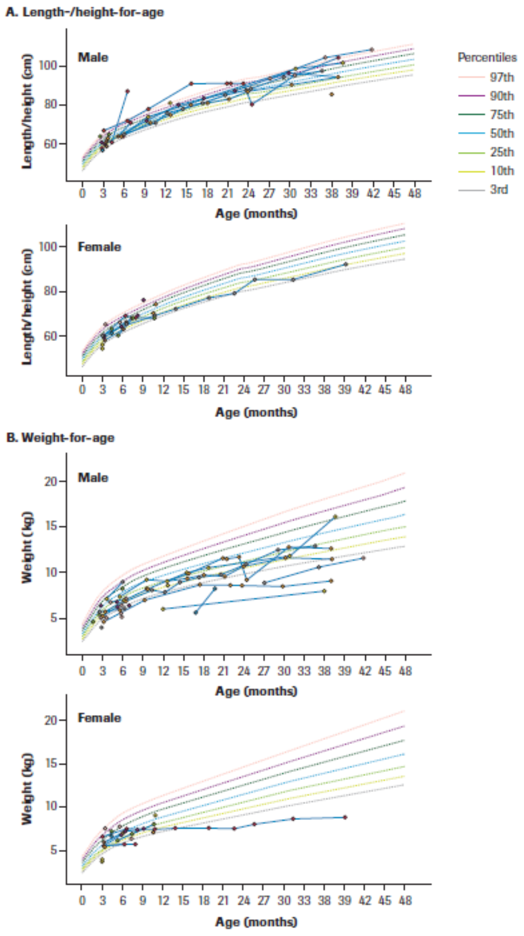

Reference lines are World Health Organization (WHO) length-/height-for-age and weight-for-age growth percentiles. For patients aged ≥ 2 years, 0.7cm is subtracted from the length to derive an estimated height, as per WHO guidelines. Patients could not stand, so the measurements were taken as lengths. All available growth measurement data are captured.

Table

Table S1. Other HINE-2 motor milestones: ANCHOVY

	M3 (N = 60)	M6 (N = 60)	M9 (N = 60)	M12 (N = 60)	M15 (N = 60)	M18 (N = 60)	M21 (N = 60)	M24 (N = 60)
**Voluntary grasp, n (%)**								
0: No grasp	**15 (25)**	**9 (15)**	**4 (7)**	**8 (13)**	**3 (5)**	**3 (5)**	**4 (7)**	**4 (7)**
1: Uses whole hand	**12 (20)**	**9 (15)**	**4 (7)**	0	**2 (3)**	**2 (3)**	0	**1 (2)**
3: Pincer grasp	0	0	**1 (2)**	0	0	0	0	0
Not recorded in chart*	3 (5)	4 (7)	0	1 (2)	0	0	0	0
**Kicking, n (%)**								
0: No kicking	**18 (30)**	**18 (30)**	**7 (12)**	**9 (15)**	**5 (8)**	**5 (8)**	**4 (7)**	**5 (8)**
1: Kicks horizontal; legs do not lift	**5 (8)**	**2 (3)**	**2 (3)**	0	0	0	0	0
2: Upward (vertical)	**2 (3)**	0	0	0	0	0	0	0
Not recorded in chart*	5 (8)	2 (3)	0	0	0	0	0	0
**Rolling, n (%)**								
0: No rolling	**22 (37)**	**18 (30)**	**8 (13)**	**9 (15)**	**5 (8)**	**5 (8)**	**4 (7)**	**5 (8)**

1: Rolling to side	**2 (3)**	**1 (2)**	0	0	0	0	0	0
Not recorded in chart*	6 (10)	3 (5)	1 (2)	0	0	0	0	0
**Crawling, n (%)**								
0: Does not lift head	**23 (38)**	**18 (30)**	**7 (12)**	**9 (15)**	**5 (8)**	**5 (8)**	**4 (7)**	**5 (8)**
Not recorded in chart*	7 (12)	4 (7)	2 (3)	0	0	0	0	0
**Standing, n (%)**								
0: Does not support weight	**24 (40)**	**18 (30)**	**7 (12)**	**9 (15)**	**5 (8)**	**5 (8)**	**4 (7)**	**5 (8)**
Not recorded in chart*	6 (10)	4 (7)	2 (3)	0	0	0	0	0
**Walking, n (%)**								
1: Bouncing	**1 (2)**[†]	0	0	0	0	0	0	0
Not recorded in chart*	28 (47)	21 (35)	8 (13)	8 (13)	5 (8)	5 (8)	4 (7)	5 (8)
Missing[‡,§]	30 (50)	34 (57)	35 (58)	26 (43)	27 (45)	27 (45)	28 (47)	27 (45)
Censored[‡,¶]	0	1 (2)	1 (2)	2 (3)	2 (3)	2 (3)	2 (3)	2 (3)
Death[‡,**]	0	3 (5)	15 (25)	23 (38)	26 (43)	26 (43)	26 (43)	26 (43)

*Hospital record exists at this time, but no mention of this motor milestone. [†]Data entry error, patient did not achieve "bouncing". [‡] Applies to voluntary grasp, kicking, rolling, crawling, standing and walking data. [§]No records for this time period. [¶]Patient was excluded from study at time points after starting treatment (nusinersen) or after enrollment in a clinical trial.

**Patient died before the beginning of this time period.

There was no consistent assessment schedule that patients followed during the course of medical care and as such, most time points have sparse data for HINE-2 assessments. After 24 months, two patients achieved voluntary grasp (using the whole hand). No other motor milestone achievements were reported.

HINE-2, Hammersmith Infant Neurological Examination, Module 2; M, month.
